# The Improving Effect and Safety of Probiotic Supplements on Patients with Osteoporosis and Osteopenia: A Systematic Review and Meta-Analysis of 10 Randomized Controlled Trials

**DOI:** 10.1155/2021/9924410

**Published:** 2021-07-24

**Authors:** Liuting Zeng, Ganpeng Yu, Kailin Yang, Wensa Hao, Hua Chen

**Affiliations:** ^1^Department of Rheumatology and Clinical Immunology, Peking Union Medical College Hospital, Chinese Academy of Medical Sciences and Peking Union Medical College, Beijing, China; ^2^People's Hospital of Ningxiang City, Ningxiang City, Hunan Province, China; ^3^Capital Medical University, Beijing, China; ^4^Institute of Materia Medica, Chinese Academy of Medical Sciences and Peking Union Medical College, Beijing, China

## Abstract

**Aim:**

Probiotics are considered to be bone metabolism regulators, and their efficacy as an adjuvant treatment option for osteoporosis is still controversial. The purpose of this study is to compare the available data from randomized controlled trials (RCT) of probiotics in the treatment of osteoporosis and osteopenia.

**Methods:**

As of June 2021, databases such as Medline, Embase, Web of Science, and Central Cochrane Library have been used for English-language literature searches and CNKI and China Biomedical Database have been used for Chinese-language literature searches. RevMan 5.3 was used for bias risk assessment, heterogeneity detection, and meta-analysis. This research has been registered in PROSPERO (CRD42020085934).

**Results:**

This systematic review and meta-analysis included 10 RCTs involving 1156. Compared with the placebo, the absolute value of lumbar spine's BMD was not statistically significant (WMD 0.04 (−0.00, 0.09), *P*=0.07, random effect model), while the percentage of lumbar spine's BMD was higher (SMD 1.16 (0.21, 2.12), *P*=0.02, random effect model). Compared with the control group, the percentage of total hip's BMD was not statistically significant (SMD 0.52 (−0.69, 1.73), *P*=0.40, random effect model). The safety analysis showed that, compared with control group, the adverse events in the experimental group were not statistically significant (RR 1.02 (0.92, 1.12), *P*=0.70, fixed effect model).

**Conclusion:**

Probiotics may be safety supplements to improve the lumbar spine's BMD of patients with osteoporosis and osteopenia. More large-sample, random-controlled, high-quality RCTs are needed to further verify the effectiveness and safety of probiotics in intervening osteoporosis or osteopenia.

## 1. Introduction

Osteoporosis is one of the diseases most closely related to the aging of the social population. It is a common bone disease characterized by bone loss and bone tissue structural degradation [1]. Osteoporotic bone loss usually has no obvious clinical manifestations in the early stage of the disease. However, as the disease progresses and bone mass is continuously lost, the bone microstructure will become more severely destroyed, and patients will have a series of clinical manifestations. For example, patients with osteoporosis can have bone pain, which can occur in the bones of the whole body, or only low back pain; when osteoporosis develops to a serious degree, hunchback and compression fractures can occur. The most serious complication of osteoporosis is osteoporotic fractures, and if such fractures have occurred, the risk of refractures increases significantly [2, 3]. The main cause of osteoporosis is that bone resorption dominated by osteoclasts is greater than bone formation dominated by osteoblasts; that is, bone remodeling has a negative balance [4, 5]. Osteoporosis is currently mainly divided into secondary osteoporosis and primary osteoporosis [6, 7]. The etiology of secondary osteoporosis is relatively clear, mainly endocrine factors, nutritional factors, disuse factors, genetic factors, immune factors, drug factors, etc. [6]. The onset of primary osteoporosis is related to heredity, aging, hormone levels, immunity, environmental factors, and nutritional status [7, 8]. According to the pathogenesis of osteoporosis, the current treatment needs to be combined with lifestyle adjustment, bone health supplement addition, drug intervention, and rehabilitation [9–11].

Recent studies have found that the intestinal flora is related to the loss of bone mass and the incidence of osteoporosis in the human body. These microorganisms may change the relative activity of osteoclasts and osteoblasts through their own metabolites, affect host metabolism and immune system, and thus affect bone metabolism. Probiotics are currently proven to have an effect on bone metabolism [12–14]. Many studies have also shown that probiotics have health-promoting effects in preventing and curing diseases. For example, probiotics can prevent or treat acute, antibiotic-related and *Clostridium-difficile*-related diarrhea [15, 16], improve inflammatory bowel disease and irritable bowel syndrome (IBS) [17, 18], reduce the risk of late-onset sepsis and necrotizing enterocolitis in newborns [19, 20], and have neuroprotective effects on neurodegenerative diseases (such as Parkinson's) [21, 22]. The same research on the treatment of osteoporosis with probiotics shows that supplementing with probiotics can prevent osteoporosis and bone loss [23, 24]. A number of clinical studies have shown that probiotics can improve the bone condition of patients with osteopenia. However, there is no systematic evaluation and summary of these clinical trials, which makes the evidence scattered and inconsistent, unable to provide new evidence for the clinic and provide new reference value for the next clinical trial design [25–35]. Therefore, this study would conduct a systematic review and meta-analysis to assess the effectiveness and safety of probiotics on postmenopausal women with osteoporosis or osteopenia for the first time, in order to provide new clinical reference information in the future.

## 2. Materials and Methods

### 2.1. Protocol

The systematic review and meta-analysis were conducted strictly in accordance with the protocol registered in PROSPERO (CRD42020085934) and PRISMA-guidelines (Supplementary Materials).

### 2.2. Selection Criteria

(1) Participants are patients who have osteoporosis or osteopenia or may suffer from osteopenia. (2) Intervention: the intervention of the experimental group is probiotics with various preparations and dosages. The intervention of the control group is a placebo or other nonprobiotic intervention methods. (3) Outcomes: primary outcomes are bone mineral density (BMD), adverse events; secondary outcomes are I collagen carboxy terminal peptide (CTX), osteoprotegerin (OPG), Receptor Activator of Nuclear Factor-*κ* B Ligand (RANKL), and Osteocalcin (OC). (4) Study design is RCTs. (5) Exclusion criteria are Non-RCT.

### 2.3. Literature Search Strategy

Web of Science, MEDLINE Complete, VIP Database for Chinese Technical Periodicals, Wanfang Database on Academic Institutions in China, PubMed, China Biology Medicine (CBM), and China National Knowledge Infrastructure (CNKI) were utilized for literature search with the retrieval time up to June 2021. The search strategy of PubMed and Embase is shown in Table S1 as an example.

### 2.4. Literature Screening

The two reviewers read independently, preliminary screening based on the title and abstract of the article, and read the full text if it is an RCT. The RCTs that meet the standards will be classified and evaluated and cross-checked by the two reviewers. When opinions differ, they will be discussed with all reviewers to decide whether to include the article.

### 2.5. Data Extraction and Risk of Bias Assessment

According to the selection criteria, data are extracted from RCTs' countries, sample size, intervention measures, baseline data, and research duration. Two evaluators independently perform data extraction, entry, and cross-check after completion to ensure data accuracy. The risk of bias of RCTs was assessed according to Cochrane Handbook for Systematic Reviews of Interventions [36]. The content of the evaluation includes random sequence generation, allocation concealments, blinding, incomplete outcomes, selective reporting, and other biases.

### 2.6. Statistical Analysis

Data analysis was performed using RevMan 5.3 statistical software provided by the Cochrane Collaboration. The measurement data use mean difference (MD) as the effect size, and the effect size is expressed in a 95% confidence interval (CI). The enumeration data are expressed by Risk Ratio (RR) and 95% CI. The *χ*2 test is used to evaluate the heterogeneity of the RCTs. When *P* ≥ 0.05 or I 2 ≤ 50%, the fixed effects model is used for analysis; otherwise, the source of heterogeneity is analyzed first, and the random effects model is used when the source of heterogeneity cannot be eliminated.

## 3. Results

### 3.1. Results of the Search

**Figure 1:**
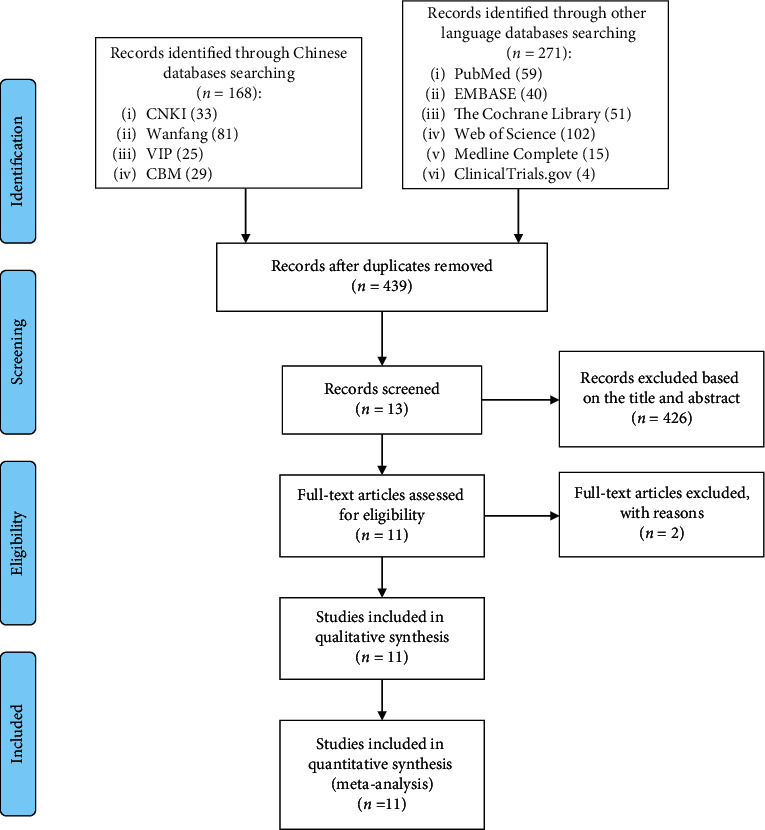
Flow diagram.

The total records identified through database searching and other sources were 439. According to the search strategy, a total of 13 articles were obtained through preliminary search. By eliminating duplicate documents, carefully reading the title and abstract, a total of 426 articles were excluded. After carefully reading the full text and comparing the selection criteria, 11 records (10 RCTs) were screened out and finally included [25–35] (Figure 1). Among the excluded research, the study by Zhang et al. did not use randomization [37].

### 3.2. Description of Included Trials

The 10 RCTs are all from different countries, and the research scale is about 40–100 participants. The intervention measures of the 10 RCTs are all probiotics, but the sources of probiotics are different. The details of study characteristics are presented in Table 1.

### 3.3. Risk of Bias of Included Studies

**Figure 2:**
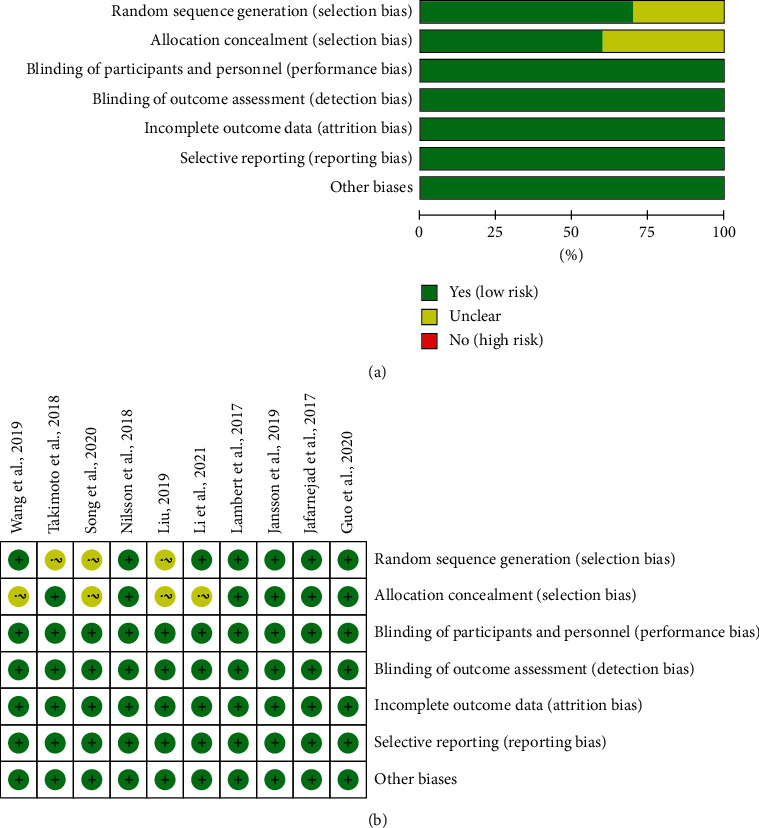
Risk of bias assessment. (a) Risk of bias graph; (b) risk of bias summary.

The summary and graph of risk of bias are shown in Figure 2.

#### 3.3.1. Sequence Generation and Allocation Concealment

Three RCTs describe their random sequence generation method: Lambert et al. and Jafarnejad et al. [26] and Jansson et al. [30] used computer-generated random numbers; Derwa et al. [18, 19] used a website (http://www.randomization.com) to generate random sequences. Li et al. [31], Wang et al. [32], and Guo et al. [33] used the random number table method to generate random numbers. Therefore, these RCTs were assessed as low risk of bias. Takimoto et al. [27], Liu [34], and Song et al. [35] did not describe the method of random sequence generation, so its risk of bias was assessed as unclear.

Lambert et al. [25], Jansson et al. [30], and Guo et al. [33] used tablets with the same taste and appearance and packaged them in identical, sealed, white cardboard boxes. The random sequence of Jafarnejad et al. [26] was generated by computer, and the researchers who recruited the subjects could not predict the distribution. The researchers and patients of Takimoto et al. [27] were not aware of the distribution during the study period. The experimental group and control group of Nilsson et al. [28, 29] used the same outer packaging. Therefore, those RCTs were considered to have implemented allocation concealment and were assessed as low risk of bias. Li et al. [31], Wang et al. [32], Liu [34], and Song et al. [35] did not mention allocation concealment, and the risk of bias was not clear.

#### 3.3.2. Blinding, Incomplete Outcome Data, and Selective Reporting

Five RCTs [25–30] describe the process of blind implementation to patients and researchers and are therefore considered to be a low risk of bias. Five RCTs [31–35] did not mention whether to use blinding, but their outcomes are objective indicators and would not be affected by not using blinding, so they are assessed as low-risk bias. Although the 10 RCTs [25–35] have missing data, the reasons for the missing and the number are balanced or utilized intention-to-treat analysis, so they are considered low risk of bias in “incomplete outcomes.” All RCTs do not have selective reporting and are therefore considered to be a low risk of bias.

#### 3.3.3. Other Potential Bias

Other sources of bias were not observed in 10 RCTs; therefore, the risks of other bias of the RCTs were low.

### 3.4. Primary Outcomes

#### 3.4.1. BMD

**Figure 3:**
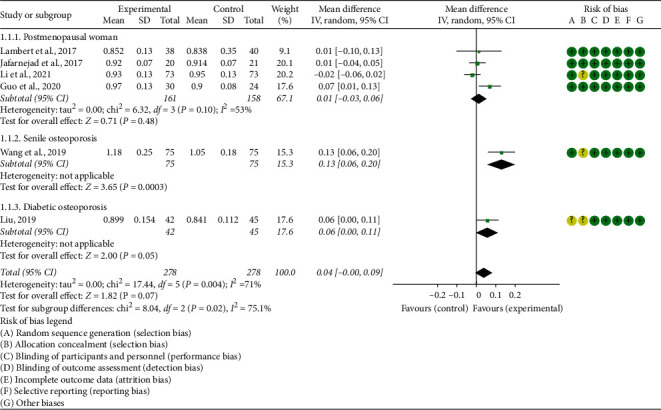
The absolute value of lumbar spine's BMD.

**Figure 4:**
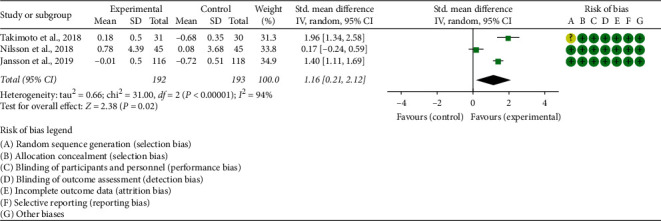
The percentage of lumbar spine's BMD improvement.

**Figure 5:**
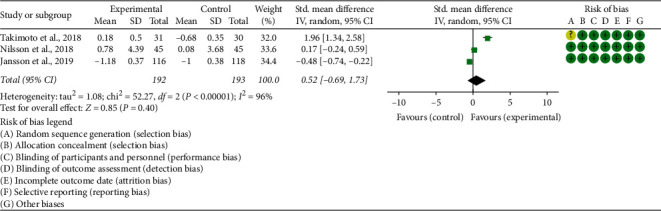
The percentage of total hip's BMD improvement.

Seven RCTs reported the absolute value of BMD, and 3 RCTs reported the percentage of BMD improvement:

1. The absolute value of lumbar spine's BMD: the heterogeneity test results showed I2 = 53% and *P*=0.10 (in postmenopausal woman), suggesting that the heterogeneity is medium, and the random effects model is used. In postmenopausal woman subgroup, the improvement of BMD in the experimental group was not statistically significant compared with the control group (WMD 0.01 (−0.03, 0.06), *P*=0.48, random effect model). In senile osteoporosis, the improvement of BMD in the experimental group was higher (WMD 0.13 (0.06, 0.20), *P*=0.0003, random effect model). In diabetic osteoporosis, the improvement of BMD in the experimental group was not statistically significant compared with the control group (WMD 0.06 (0.00, 0.11), *P*=0.05, random effect model) (Figure 3). The summary results also showed that the improvement of BMD in the experimental group was not statistically significant compared with the control group (WMD 0.04 (−0.00, 0.09), *P*=0.07, random effect model).
2. The percentage of lumbar spine's BMD improvement: since the data unit of this indicator is not uniform, standardized MD (SMD) is used for analysis. The heterogeneity test results showed I2 = 94% and *P* < 0.00001, suggesting that the heterogeneity is high, and the random effects model was used. The summary results showed that, compared with the control group, the improvement of BMD in the experimental group was higher (SMD 1.16 (0.21, 2.12), *P*=0.02, random effect model) (Figure 4).
3. The percentage of total hip's BMD improvement: since the data unit of this indicator is not uniform, standardized MD (SMD) is used for analysis. The heterogeneity test results showed I2 = 96% and *P* < 0.00001, suggesting that the heterogeneity is high, and the random effects model is used. The summary results showed that, compared with the control group, the improvement of BMD in the experimental group was of no statistical significance (SMD 0.52 (−0.69, 1.73), *P*=0.40, random effect model) (Figure 5).
4. Jafarnejad et al. [26] reported the absolute value of forearm BMD; they found that the improvement of total hip's BMD in the experimental group was not statistically significant compared with the control group (*P*=0.725). Lambert et al. [25] reported the absolute value of femoral neck and trochanter's BMD; they found that compared with control group, the improvement of BMD in the experimental group was higher (femoral neck: *P*=0.0059; trochanter: *P*=0.03). Song et al. [35] reported the absolute value of total hip's BMD. It showed that, compared with the control group, the BMD of both forearms improved significantly (*P* < 0.05).

### 3.5. Secondary Outcomes

#### 3.5.1. CTX

**Figure 6:**
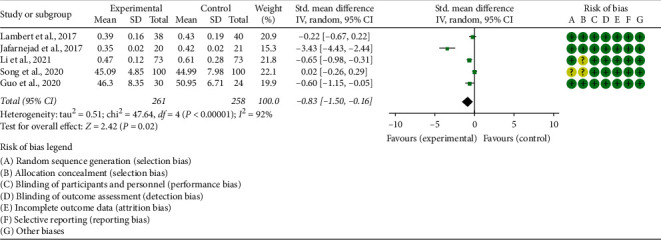
The results of CTX.

Five RCTs reported CTX. The heterogeneity test results showed I2 = 92% and *P* < 0.00001, suggesting that the heterogeneity is high, and the random effects model is used. The summary results showed that, compared with the control group, the CTX in the experimental group was lower (SMD −0.83 (−1.50, −0.16), *P*=0.02, random effect model) (Figure 6).

#### 3.5.2. OPG and RANKL

**Figure 7:**
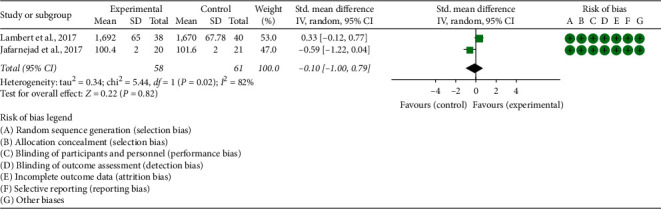
The results of OPG.

**Figure 8:**
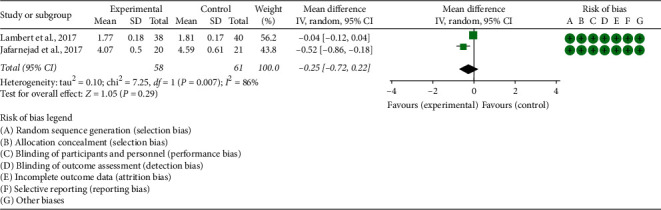
The results of RANKL.

Two RCTs reported OPG and RANKL. (1) OPG: the heterogeneity test results showed I2 = 82% and *P*=0.02, suggesting that the heterogeneity is high, and the random effects model is used. The summary results showed that the improvement of OPG in the experimental group was not statistically significant compared with the control group (WMD −0.10 (−1.00, 0.79), *P*=0.82, random effect model) (Figure 7). (2) RANKL: the heterogeneity test results showed I2 = 86% and *P*=0.007, suggesting that the heterogeneity is high, and the random effects model is used. The summary results showed that the improvement of RANKL in the experimental group was not statistically significant compared with the control group (SMD −0.25 (−0.72, 0.22), *P*=0.29, random effect model) (Figure 8).

#### 3.5.3. OC

**Figure 9:**
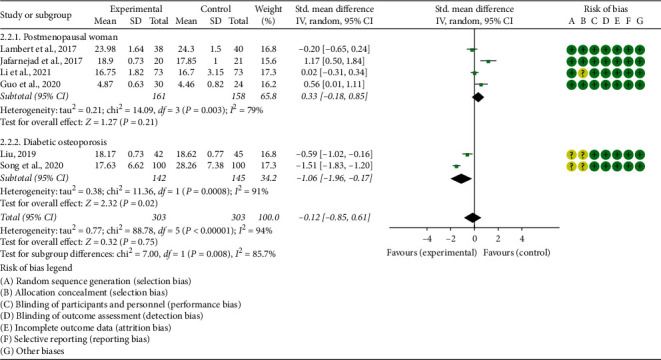
The results of OC.

Six RCTs reported OC. The heterogeneity test results showed that (1) in postmenopausal women subgroup: I2 = 79% and *P*=0.003; (2) in diabetic osteoporosis subgroup, I2 = 91% and *P*=0.0008, suggesting that the heterogeneity is high, and the random effects model is used. In postmenopausal women subgroup, the improvement of OC in the experimental group was not statistically significant compared with the control group (SMD 0.33 (−0.18, 0.85), *P*=0.21, random effect model). In diabetic osteoporosis subgroup, the OC in the experimental group was lower (SMD −1.06 (−1.96, −0.17), *P*=0.02, random effect model). The summary results showed that the improvement of OC in the experimental group was not statistically significant compared with the control group (SMD −0.12 (−0.85, 0.61), *P*=0.75, random effect model) (Figure 9).

### 3.6. Adverse Events

**Figure 10:**
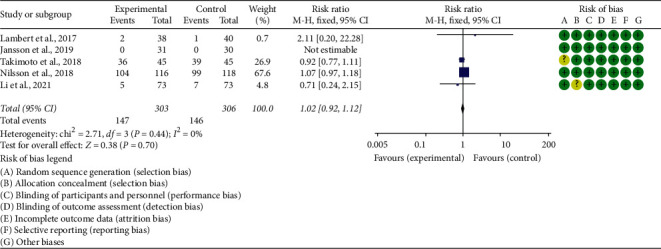
Adverse events.

Five RCTs reported the adverse events. The heterogeneity test results showed I2 = 0% and *P*=0.44, suggesting that the heterogeneity is low, and the fix effects model is used. The summary results showed that the adverse events in the experimental group were not statistically significant compared with the control group (RR 1.02 (0.92, 1.12), *P*=0.70, fixed effect model) (Figure 10).

## 4. Discussion

In this paper, the clinical studies of probiotics intervention for osteopenia mostly use radiographical indicators (BMD) and biochemical indicators (CTX, OPG, RANKL, OC), which are more objective. Therefore, the efficacy criteria can be considered reliable. The 10 studies included in this study are all RCTs of different preparations of probiotics that interfere with osteoporosis or osteopenia. The results of the meta-analysis showed that, compared with the control group, the difference in some of the primary outcomes was statistically significant, suggesting that probiotics have a certain effect on antiosteoporosis. The specific results are (1) in postmenopausal woman, compared with control group; the improvement of absolute value of lumbar spine's BMD was of no statistical significance, but that of the percentage of lumbar spine's BMD was higher. In senile osteoporosis, the improvement of absolute value of lumbar spine's BMD in experimental group was higher, while in diabetic osteoporosis, that was of no statistical significance. (2) Compared with control group, the improvement of absolute value and percentage of total hip's BMD was of no significance in postmenopausal woman. (3) The CTX level in the experimental group was lower. In postmenopausal women subgroup, the improvement of OC in the experimental group was not statistically significant; however, in diabetic osteoporosis subgroup, the OC in the experimental group was lower. This suggest that probiotics have bone protection. (4) Compared with control group, there was no statistical difference in the changes of OPG, RANKL in the experimental group. (5) The incidence of adverse events in the experimental group was not statistically different from that in the control group, suggesting that the use of probiotics would not increase the incidence of adverse events.

In terms of improving lumbar spine BMD in postmenopausal woman, the improvement of its absolute value is different from that of its percentage, and the heterogeneity between RCTs is medium. Since those RCTs are from different countries, we suspect that the main reason for this result may be related to the different reactions of different nationalities to probiotics. This study also showed that, in postmenopausal woman, probiotics have an improvement effect on lumbar spine BMD, but the improvement effect on total hip BMD is not obvious. This suggests that probiotics have different effects on the bones of different parts of postmenopausal women. However, further long-term studies are needed to explore the obvious bone site-specific effects of probiotic treatment on postmenopausal women. The RCT on diabetic osteoporosis also showed that probiotics can improve the BMD of the forearm, but the improvement of the lumbar spine BMD is not obvious. However, because diabetic osteoporosis involves only 1–2 RCTs, more relevant RCTs are needed to further verify or modify the conclusion. In addition, although these RCTs utilized probiotics, the bacterial species used in each study are different, so this difference may also be related to the different bacterial species. Among the biochemical indicators OPG, RANKL, and OC, the heterogeneity between RCTs is relatively large, which may be related to individual differences. However, due to the fact that there are fewer RCTs including probiotics to interfere with osteoporosis and osteopenia and the control group is placebo, more RCTs with large samples, uniform probiotic preparation, and different control drugs are needed to verify the improvement effect of probiotics on osteoporosis or bone loss. In the incidence of adverse events, there was no statistical difference between the probiotic group and the control group. Limited to the number of included RCTs, there is no enough evidence to verify the incidence of adverse events in treatment of probiotics and whether probiotics combined with antiosteoporosis drugs can reduce the incidence of adverse events. The occurrence of adverse events is often related to drugs and treatment methods. Because most patients with osteoporosis and osteopenia are elderly patients, they often have other medical conditions. Therefore, while applying probiotics to treat osteoporosis, in addition to choosing the right treatment, it is also necessary to take preventive measures against possible adverse events.

Osteoporosis is an epidemic metabolic bone disease characterized by bone loss and structural destruction [38], which easily leads to fractures and disability. It is affected by a variety of genetic factors and environmental factors, such as genetics, diet, hygiene, and the use of antibiotics. With the aging of the population becoming more and more serious, there are more than 200 million people in the world suffering from osteoporosis, and its incidence has jumped to the sixth place among common and frequently-occurring diseases, becoming a global public health problem [39]. Among the population with osteoporosis, postmenopausal women are the majority. The lack of estrogen in postmenopausal women increases their risk of osteoporosis. Postmenopausal osteoporosis not only has a high incidence, but also has serious complications. It has always been the focus of prevention and treatment [40].

Current studies have found that the intestinal flora is related to the loss of bone mass and the incidence of osteoporosis in the human body. These microorganisms may change the relative activity of osteoclasts and osteoblasts through their own metabolites, affecting host metabolism and immune system, thereby affecting bone metabolism [41]. Used in an appropriate amount, probiotics have been shown to change and synthesize the metabolites of the intestinal flora [41] and regulate the immune response in the host [42, 43]. Importantly, probiotics can enhance the epithelial barrier function. These effects explain the beneficial effects of probiotics [44, 45]. Among them, *Lactobacillus acidophilus* is a kind of *Lactobacillus*. After fermentation in the intestine, it can produce lactic acid, butyric acid, and acetic acid, which can improve the utilization of calcium, phosphorus, and iron and promote the absorption of iron and vitamin D [46]. Another widely studied probiotic is *Lactobacillus rhamnosus* (LGG), which also belongs to the genus Lactobacillus, a third-generation probiotic. Current research reports show that *Lactobacillus rhamnosus* can prevent bone loss induced by ovariectomy, reduce intestinal permeability, and improve intestinal and systemic inflammation [47].

In addition, the intestinal flora is considered a virtual “endocrine organ” because it affects host hormone levels. And because some bacteria can produce and secrete hormones, including serotonin and dopamine, and sex hormones, they may regulate bone remodeling by affecting hormone levels [48]. In particular, for the treatment of postmenopausal osteoporosis, intestinal flora, especially intestinal probiotics, has been proven to be a potential therapeutic strategy [49]. Among them, *Lactobacillus rhamnosus* can stimulate bone formation by increasing estrogen [50]. The study found that prebiotics can increase the number of probiotics such as lactobacilli and butyric acid bacteria to promote the secretion of more short-chain fatty acids, thereby reducing the intestinal PH value and increasing the solubility of calcium in the intestinal lumen, thus increasing the bone mineral content and bone mineral density of young people [51]. Prebiotics are indigestible and fermentable food ingredients that not only promote the growth of intestinal probiotics, but also promote the production of probiotic metabolites [52], and a variety of substrates that can be metabolized by bacteria besides sugars [53]. Many studies have proven that prebiotics enhance human calcium absorption [54].

Compared with previous systematic review and meta-analysis [55], this study is the newest systematic review and meta-analysis strictly based on the PRISMA guidelines with preregistered plans. This study also covers a wider population (postmenopausal women, senile osteoporosis, and diabetic osteoporosis) than previous reviews. Meanwhile, this study included 7 RCTs about postmenopausal women involving 719 participants; the applicable population of the conclusion has also been extended to the East Asian population. This study also found that in the same population, probiotics can improve the BMD of different body parts differently. For different groups of people, the improvement of BMD of the same body part by probiotics is also different. These may inspire future research. The advantage of this study is that this is the first systematic review and meta-analysis of RCTs in which probiotics interfere with osteoporosis or osteopenia. The disadvantage is that the results may be affected due to the lack of included RCTs: (1) because different studies use different probiotics as clinical interventions, they may be interest related, and this inconsistency may certainly affect the strength of the argument. (2) Although RCTs come from different countries, they are all single-center, small-sample clinical trials.

## 5. Conclusion

Probiotics may improve BMD and reduce CTX and OC, but there is no difference in improving OPG and RANKL. This may be due to the small number of included RCTs and the influence of many factors, and further research is needed. The incidence of adverse events in the probiotic group is comparable to that in the control group and can be considered a safe intervention. In future research, we should pay attention to the standardization of clinical research evidence-based medicine methodology and optimize clinical treatment plans. In the future, more large-sample, random-controlled, high-quality RCTs are needed to further verify the effectiveness and safety of probiotics in intervening osteoporosis or osteopenia.

## Figures and Tables

**Table 1:** The characteristics of the included studies.

Study	Country	Participant	Sample size		Intervention		Relevant outcomes	Mean age (years)		BMI		Duration
			Trial group	Control group	Trial group	Control group		Trial group	Control group	Trial group	Control group
Lambert et al. [25]	Denmark	Postmenopausal osteopenic women	38	40	Red clover extract (RCE) (rich in isoflavone aglycones and probiotics)	Placebo [made by 90 L of water mixed with 250 g brown food coloring (ammoniated caramel) (Kavli)]	BMD, CTX, OPG, RANKL, OC, adverse events	60.84 ± 1.07	62.85 ± 0.99	24.84 ± 0.62	26.65 ± 0.81	12 months

Jafarnejad et al. [26]	Iran	Postmenopausal osteopenic women	20	21	Multispecies probiotic supplement (GeriLact capsule)	Placebo	BMD, CTX, RANKL, OPG, OC	58.85 ± 0.68	57.29 ± 0.72	24.86 ± 0.41	23.82 ± 0.38	6 months

Takimoto et al. [27]	Japan	Healthy postmenopausal women	31	30	Probiotic *Bacillus subtilis* C-3102 (C-3102)	Placebo	BMD, adverse events	57.5 ± 4.3	57.8 ± 5.4	22.2 ± 3.3	22.1 ± 2.7	6 months

Nilsson et al. [28, 29]	Sweden	Postmenopausal women with low bone mineral density	45	45	Freeze-dried *L. reuteri* 6475 (BioGaia AB, Stockholm, Sweden)	Placebo (maltodextrin powder)	BMD, adverse events	76.4 ± 1.0	76.3 ± 1.1	25.5 ± 3.5	25.3 ± 3.3	12 months

Jansson et al. [30]	Sweden	Healthy postmenopausal women	126	123	Three lactobacillus strains (*L. paracasei* 8700 : 2 (DSM 13434), *L. plantarum* heal 9 (DSM 15312), and *L. plantarum* heal 19 (DSM 15313))	Placebo	BMD, adverse events	59.1 ± 3.8	58.1 ± 4.3	24.2 ± 2.7	23.9 ± 2.6	12 months

Li et al. [31]	China	Postmenopausal osteopenic women	73	73	Bifidobacterium quadruple viable bacteria tablets 0.5 g Tid + oral alendronate sodium 10 mg Qd + subcutaneous or intramuscular injection of salmon calcitonin 50 IU Qd.	Oral alendronate sodium 10 mg Qd + subcutaneous or intramuscular injection of salmon calcitonin 50 IU Qd.	BMD, CTX, OC, adverse events	68.15 ± 22.36	69.82 ± 21.47	26.31 ± 8.36	24.85 ± 7.40	6 months

Wang et al. [32]	China	Senile osteoporosis	75	75	Bifidobacterium triple live bacteria capsules 840 mg Bid + conventional therapy	Conventional therapy	BMD	71.52 ± 5.46	71.68 ± 5.41	—	—	2 months

Guo [33]	China	Postmenopausal osteopenic women	30	24	Dry Probio-M8 lactic acid bacteria	Placebo	BMD, CTX, OC	61.91 ± 6.37	6.34 ± 5.71	23.59 ± 3.43	23.86 ± 3.19	6 months

Liu [34]	China	Diabetic osteoporosis	42	45	Bifidobacterium triple viable enteric-coated capsules + conventional therapy	Conventional therapy	BMD, OC	70.5 ± 6.8	69.8 ± 6.4	—	—	6 months

Song et al. [35]	China	Diabetic osteoporosis	100	100	Quadruple bifidobacterium live bacteria tablets + conventional therapy	Conventional therapy	BMD, CTX, OC	68.20 ± 12.78	69.76 ± 12.09	25.21 ± 2.55	26.90 ± 2.39	12 months

## Data Availability

All data generated or analyzed during this study are included in this article.

## Supplementary Materials

Supplementary Materials PRISMA 2009 checklist: checklist; Table S1: search Strategies for PubMed and Embase.

## References

[1] Baccaro L. F., Conde D., Costa-Paiva L., Pinto-Neto A. M. (2015). The epidemiology and management of postmenopausal osteoporosis: a viewpoint from Brazil. *Clinical Interventions in Aging*.

[2] Lane J. M., Russell L., Khan S. N. (2000). Osteoporosis. *Clinical Orthopaedics and Related Research*.

[3] Khan A., Fortier M., Fortier M. (2014). Osteoporosis in menopause. *Journal of Obstetrics and Gynaecology Canada*.

[4] Armas L. A. G., Recker R. R. (2012). Pathophysiology of osteoporosis. *Endocrinology and Metabolism Clinics of North America*.

[5] Cymet T. C., Wood B., Orbach N. (2000). Osteoporosis. *The Journal of the American Osteopathic Association*.

[6] Eastell R., Szulc P. (2017). Use of bone turnover markers in postmenopausal osteoporosis. *The Lancet Diabetes & Endocrinology*.

[7] Muhammad A., Mada S. B., Malami I. (2018). Postmenopausal osteoporosis and breast cancer: the biochemical links and beneficial effects of functional foods. *Biomedicine & Pharmacotherapy*.

[8] Kerschan-Schindl K. (2016). Prevention and rehabilitation of osteoporosis. *Wiener Medizinische Wochenschrift*.

[9] Miller P. D. (2016). Management of severe osteoporosis. *Expert Opinion on Pharmacotherapy*.

[10] Gatti D., Fassio A. (2019). Pharmacological management of osteoporosis in postmenopausal women: the current state of the art. *Journal of Population Therapeutics and Clinical Pharmacology*.

[11] Kanis J. A., Cooper C., Cooper C., Rizzoli R., Reginster J.-Y. (2019). European guidance for the diagnosis and management of osteoporosis in postmenopausal women. *Osteoporosis International*.

[12] Zhong Y., Zheng C., Zheng J. H., Xu S. C. (2020). The relationship between intestinal flora changes and osteoporosis in rats with inflammatory bowel disease and the improvement effect of probiotics. *European Review for Medical and Pharmacological Sciences*.

[13] Quach D., Britton R. A. (2017). Gut microbiota and bone health. *Advances in Experimental Medicine and Biology*.

[14] Biver E., Berenbaum F., Valdes A. M. (2019). Gut microbiota and osteoarthritis management: an expert consensus of the European society for clinical and economic aspects of osteoporosis, osteoarthritis and musculoskeletal diseases (ESCEO). *Ageing Research Reviews*.

[15] Goldenberg J. Z., Yap C., Lytvyn L. (2017). Probiotics for the prevention of Clostridium difficile-associated diarrhea in adults and children. *Cochrane Database of Systematic Reviews*.

[16] Ma Y., Yang J. Y., Peng X., Xiao K. Y., Xu Q., Wang C. (2020). Which probiotic has the best effect on preventing Clostridium difficile ‐associated diarrhea? A systematic review and network meta‐analysis. *Journal of Digestive Diseases*.

[17] Abraham B. P., Quigley E. M. M. (2017). Probiotics in inflammatory bowel disease. *Gastroenterology Clinics of North America*.

[18] Derwa Y., Gracie D. J., Hamlin P. J., Ford A. C. (2017). Systematic review with meta-analysis: the efficacy of probiotics in inflammatory bowel disease. *Alimentary Pharmacology & Therapeutics*.

[19] Costeloe K., Bowler U., Brocklehurst P. (2016). A randomised controlled trial of the probiotic Bifidobacterium breve BBG-001 in preterm babies to prevent sepsis, necrotising enterocolitis and death: the Probiotics in Preterm infantS (PiPS) trial. *Health Technology Assessment*.

[20] Bi L.-W., Yan B.-L., Yang Q.-Y., Li M.-M., Cui H.-L. (2019). Probiotic strategies to prevent necrotizing enterocolitis in preterm infants: a meta-analysis. *Pediatric Surgery International*.

[21] Alipour Nosrani E., Tamtaji O. R., Alibolandi Z. (2021). Neuroprotective effects of probiotics bacteria on animal model of Parkinson’s disease induced by 6-hydroxydopamine: a behavioral, biochemical, and histological study. *Journal of Immunoassay and Immunochemistry*.

[22] Tamtaji O. R., Milajerdi A., Reiner Ž. (2020). A systematic review and meta-analysis: the effects of probiotic supplementation on metabolic profile in patients with neurological disorders. *Complementary Therapies in Medicine*.

[23] Collins F. L., Rios-Arce N. D., Schepper J. D., Parameswaran N., McCabe L. R. (2017). The potential of probiotics as a therapy for osteoporosis. *Microbiology Spectrum*.

[24] Paccou J. (2020). Nutritional facets of osteoporosis management: can probiotics help?. *Joint Bone Spine*.

[25] Lambert M. N. T., Thybo C. B., Lykkeboe S. (2017). Combined bioavailable isoflavones and probiotics improve bone status and estrogen metabolism in postmenopausal osteopenic women: a randomized controlled trial. *The American Journal of Clinical Nutrition*.

[26] Jafarnejad S., Djafarian K., Fazeli M. R., Yekaninejad M. S., Rostamian A., Keshavarz S. A. (20175). Effects of a multispecies probiotic supplement on bone health in osteopenic postmenopausal women: a randomized, double-blind, controlled trial. *Journal of the American College of Nutrition*.

[27] Takimoto T., Hatanaka M., Hoshino T. (2018). Effect of Bacillus subtilis C-3102 on bone mineral density in healthy postmenopausal Japanese women: a randomized, placebo-controlled, double-blind clinical trial. *Bioscience of Microbiota, Food and Health*.

[28] Nilsson A. G., Sundh D., Bäckhed F., Lorentzon M. (2018). Lactobacillus reuterireduces bone loss in older women with low bone mineral density: a randomized, placebo-controlled, double-blind, clinical trial. *Journal of Internal Medicine*.

[29] Li P., Sundh D., Ji B. (2021). Metabolic alterations in older women with low bone mineral density supplemented with Lactobacillus reuteri. *JBMR Plus*.

[30] Jansson P.-A., Curiac D., Lazou Ahrén I. (2019). Probiotic treatment using a mix of three Lactobacillus strains for lumbar spine bone loss in postmenopausal women: a randomised, double-blind, placebo-controlled, multicentre trial. *The Lancet Rheumatology*.

[31] Li F., Gong C. L., Wang Q. L., Chen Q., Lu Y. (2021). Effects of probiotics on bone metabolism in postmenopausal patients with osteoporosis. *Chinese Journal of Gerontology*.

[32] Wang X. S., Liu L., Liu H. M. (2019). Effects of probiotics on bone biochemical metabolism and osteoporosis in the elderly. *Modern Digestion and Interventional Diagnosis and Treatment*.

[33] Guo Z. G. (2020). *Clinical Study on the Effect of Probiotic Lactic Acid Bacteria on Osteoporosis in Postmenopausal Women*.

[34] Liu H. D. (2019). Observation of the efficacy of bifidobacterium triple viable enteric-coated capsules in the treatment of type 2 diabetes with osteoporosis. *Journal of Gannan Medical College*.

[35] Song Y. P., Guo Y. X., Li J. P. (2020). Supplementation of Bifidobacterium quadruple viable tablets on the effects of bone metabolism in patients with diabetic osteoporosis. *Chinese Journal of Osteoporosis*.

[36] Deeks J. J., Higgins J. P., Altman D. G., Higgins J. P., Green S. (2020). 2020: chapter 8: assessing risk of bias in included studies. *Cochrane Handbook or Systematic Reviews of Interventions Version 6.1.0*.

[37] Zhang J., Zhong J., Nie D., Lin Z., Gao S. (2018). The effect of probiotics on the improvement of bone status in postmenopausal women with osteopenia. *Chinese Journal of Microecology*.

[38] Aspray T. J., Hill T. R. (2019). Osteoporosis and the ageing skeleton. *Subcellular Biochemistry*.

[39] Sözen T., Ozisik L., Calik Basaran N. (2017). An overview and management of osteoporosis. *European Journal of Rheumatology*.

[40] Black D. M., Rosen C. J. (2016). Postmenopausal osteoporosis. *New England Journal of Medicine*.

[41] D’Amelio P., Sassi F. (2018). Gut microbiota, immune system, and bone. *Calcified Tissue International*.

[42] Scholz-Ahrens K. E., Ade P., Marten B. (2007). Prebiotics, probiotics, and synbiotics affect mineral absorption, bone mineral content, and bone structure. *The Journal of Nutrition*.

[43] Yan F., Polk D. B. (2011). Probiotics and immune health. *Current Opinion in Gastroenterology*.

[44] Anderson R. C., Cookson A. L., McNabb W. C. (2010). Lactobacillus plantarum MB452 enhances the function of the intestinal barrier by increasing the expression levels of genes involved in tight junction formation. *BMC Microbiology*.

[45] Seth A., Yan F., Polk D. B., Rao R. K. (2008). Probiotics ameliorate the hydrogen peroxide-induced epithelial barrier disruption by a PKC- and MAP kinase-dependent mechanism. *American Journal of Physiology-Gastrointestinal and Liver Physiology*.

[46] Anjum N., Maqsood S., Masud T., Ahmad A., Sohail A., Momin A. (2014). Lactobacillus acidophilus: characterization of the species and application in food production. *Critical Reviews in Food Science and Nutrition*.

[47] Li J.-Y., Chassaing B., Tyagi A. M. (2016). Sex steroid deficiency-associated bone loss is microbiota dependent and prevented by probiotics. *Journal of Clinical Investigation*.

[48] Bron P. A., van Baarlen P., Kleerebezem M. (2012). Emergingmolecular insights into the interaction between probiotics and the host intestinal mucosa. *Nature Reviews Microbiology*.

[49] Britton R. A., Irwin R., Quach D. (2014). Reuteri treatment prevents bone loss in a menopausal ovariectomized mouse model. *Journal of Cellular Physiology*.

[50] Chen C., Dong B., Wang Y. (2020). The role of Bacillus acidophilus in osteoporosis and its roles in proliferation and differentiation. *Journal of Clinical Laboratory Analysis*.

[51] Abrams S. A., Griffin I. J., Hawthorne K. M. (2005). A combination of prebiotic short- and long-chain inulin-type fructans enhances calcium absorption and bone mineralization in young adolescents. *The American Journal of Clinical Nutrition*.

[52] Roberfroid M., Gibson G. R., Hoyles L. (2010). Prebiotic effects: metabolic and health benefits. *British Journal of Nutrition*.

[53] Bindels L. B., Delzenne N. M., Cani P. D. (2015). Towards a more comprehensive concept for prebiotics. *Nature Reviews Gastroenterology & Hepatology*.

[54] Macfarlane S., Macfarlane G. T., Cummings J. H. (2006). Review article: prebiotics in the gastrointestinal tract. *Alimentary Pharmacology and Therapeutics*.

[55] Yu J., Cao G., Yuan S., Luo C., Yu J., Cai M. (2021). Probiotic supplements and bone health in postmenopausal women: a meta-analysis of randomised controlled trials. *BMJ Open*.

