# Supplementary material for: The Improving Effect and Safety of Probiotic Supplements on Patients with Osteoporosis and Osteopenia: A Systematic Review and Meta-Analysis of 10 Randomized Controlled Trials
Source: Evid Based Complement Alternat Med. 2021 Jul 24;2021:9924410. doi: 10.1155/2021/9924410 (PMC8328694; doi:10.1155/2021/9924410)
Supplement: Supplementary Materials — PRISMA 2009 checklist: checklist; Table S1: search Strategies for PubMed and Embase. [file 9924410.f1.zip › 9924410.f1/Table S1. Search Strategies for Pubmed and Embase.pdf]

Table S1. Search Strategies for Pubmed and Embase

**PubMed**	(Probiotics)<br>AND<br>(Osteoporoses OR Osteoporosis, Post-Traumatic OR Osteoporosis, Post Traumatic OR Post-Traumatic Osteoporoses OR Post-Traumatic Osteoporosis OR Osteoporosis, Senile OR Osteoporoses, Senile OR Senile Osteoporoses OR Osteoporosis, Involutional OR Senile Osteoporosis OR Osteoporosis, Age-Related OR Osteoporosis, Age Related OR Bone Loss, Age-Related OR Age-Related Bone Loss OR Age-Related Bone Losses OR Bone Loss, Age Related OR Bone Losses, Age-Related OR Age-Related Osteoporosis OR Age Related Osteoporosis OR Age-Related Osteoporoses OR Osteoporoses, Age-Related OR Metabolic Bone Diseases OR Bone Disease, Metabolic OR Metabolic Bone Disease OR Osteopenia OR Osteopenias OR Low Bone Density OR Bone Density, Low OR Low Bone Densities OR Low Bone Mineral Density)<br>AND<br>(random* controlled trial [pt] OR controlled clinical trial* [pt] OR randomized [tiab] OR placebo [tiab] OR drug therapy [sh] OR random* [tiab] OR trial* [tiab] OR group* [tiab])<br>NOT<br>(animals [mh] NOT humans [mh])
**EMBASE**	1 ' Probiotics'<br>2 ' Osteoporoses'/exp<br>3 'Post-Traumatic Osteoporoses' or 'Post-Traumatic Osteoporosis'<br>4 'Senile Osteoporoses' or 'Senile Osteoporosis'<br>5 'Age-Related Bone Loss' or 'Age-Related Bone Losses'<br>6 'Age-Related Osteoporosis' or 'Age Related Osteoporosis' or 'Age-Related Osteoporoses'<br>7 2 or 3 or 4 or 5 or 6<br>8 'Bone Diseases, Metabolic'/exp<br>9 'Metabolic Bone Diseases'<br>10 'Metabolic Bone Disease'<br>11 'Osteopenia' or 'Osteopenias'<br>12 'Low Bone Density' or 'Low Bone Densities' or 'Low Bone Mineral Density'<br>13 8 or 9 or 10 or 11 or 12<br>14 7 or 13<br>15 'randomized controlled trial'<br>16 'single blind procedure' or 'double blind procedure'<br>17 'crossover procedure'<br>18 15 or 16 or 17<br>19 1 and 14<br>20 19 and 18
